# Better Ask Than Tell: Responses to mHealth Interrogative Reminders and Associations With Colorectal Cancer Screening Subsequent Uptake in a Prospective Cohort Intervention

**DOI:** 10.2196/mhealth.9351

**Published:** 2019-01-21

**Authors:** Lea Hagoel, Nili Stein, Gad Rennert, Efrat Neter

**Affiliations:** 1 Department of Community Medicine and Epidemiology Carmel Medical Center Haifa Israel; 2 Faculty of Medicine Technion Haifa Israel; 3 Department of Behavioral Sciences Ruppin Academic Center Emeq Hefer Israel

**Keywords:** adherence, colorectal cancer, cancer screening, health behaviors, interrogative reminders, short message service text messages

## Abstract

**Background:**

Text message (short message service, SMS) interrogative reminders were adopted in population screening for the early detection of colorectal cancer (CRC).

**Objective:**

This study aims to examine responses to text message (SMS) reminders and associate responses with senders’ characteristics, message type (interrogative/declarative), and subsequent screening uptake.

**Methods:**

We conducted a prospective cohort intervention. Text message (SMS) reminders to undergo CRC screening, randomized into interrogative and declarative phrasing, were sent to nonadherent 40,000 women and men (age 50-74 years) at CRC average risk. We analyzed recipient responses by message phrasing, recipient characteristics, and for content, the latter predicting subsequent CRC screening per program database.

**Results:**

While interrogative text message (SMS) reminders elicited 7.67% (1475/19,227) responses, declarative ones elicited 0.76% (146/19,262) responses. Text message (SMS) responses were content analyzed and grouped into attitudes toward CRC screening (1237/1512, 81.8% positive) and intention to screen (1004/1512, 62.6%). Text message (SMS) respondents screened significantly more than nonrespondents after 6 months (415/1621, 25.6% vs 3322/36,868, 9.0%; χ12=487.5, *P*<.001); 1 year (340/1621, 21.0% vs 4711/36,868; χ12=91.5, *P*<.001); and 2 years (225/1621, 13.9% vs 3924/36,868; χ12=16.9, *P*<.001) following the reminders. In a multivariable logistic regression among text message (SMS) respondents, screening after 6 months was significantly predicted by older age, past sporadic screening, attitudes, and intentions.

**Conclusions:**

Interrogative text message (SMS) reminders reached previously uninvolved sectors in the CRC target population—men, sporadic-screenees, and the “never-tested” before. This novel application resulted in a population-level, incrementally enhanced screening. Asking patients about their future health behavior may be relevant for enhancing other health behaviors in preventive medicine and clinical settings.

## Introduction

The mortality rate from colorectal cancer (CRC) may be reduced following routine screening and early detection of the disease [1]. CRC screening rates, however, remain relatively low, and enhancement efforts result in a slow, cumulative, change [2]. Adherence to CRC screening is observed mainly among women and older adults; further interventions are also needed for men and younger individuals. Recommendations for innovative approaches to increase CRC screening rates advocate maintaining a “human connection” [3] with individuals in the target population. Reminders using an mobile health (mHealth) technique with attention to wording exemplify such an undertaking [4].

Short message service (SMS) text messaging emerged in 1992, and by 1995, it was a socially acceptable and widely used means of communication [3,5,6]. Since then, SMS text messaging graduated from a personal means of communication among friends and colleagues to a tool used by organizations to contact and inform target audiences [7,8]. SMS has been used (as pre- or postnotification reminders) in the health domain to improve response rates to mailed questionnaires [9], enhance appointment attendance [10,11], reduce posttreatment risk [12], adhere to medication [13], and promote self-management and risk reduction among patients with cardiovascular and coronary heart disease [14,15]. Some reviews indicated that SMS interventions are a robust means for effectively targeting health behavior changes; however, effects have been small to moderate [13,15].

A refined view of SMS is continuously evolving. Studies using the SMS method to remind individuals of recommended health behaviors often imply that this is a unidirectional communication channel. However, 2-way communication between a public agency and stakeholders has also been examined previously [16,17]; the authors inferred that SMS reminders enhanced dynamic feedback and change in health behaviors [17] and provided “information comparable to other modes” [18]. Moreover, the SMS use evoked a social context among recipients that was based on the rapport previously established between provider and health care target audiences; such a rapport is essential for long-term behavior changes [3] in health programs, including cancer early detection.

To date, while few studies have examined the effect of SMS text messages to promote participation in cancer screening [19], very few have focused on SMS text message wording for enhanced screening participation, which is important for reducing CRC-related mortality [2]. This study offers a novel, combined approach to enhance cancer screening through (1) minimal SMS text message reminders for routine CRC screening tests; (2) interrogative wording as reminders, translating a psychological technique to preventive medicine; (3) content analysis of addressees’ responses as an interactive dimension; and (4) an objective outcome measure (test performance).

This study branched out of a 50,000 participant field experiment [4], which adapted the question-behavior-effect (QBE) [20] to the population level by using an mHealth tool. Reminders (to screen) were worded as questions or as statements, and either invoked a social comparison or not [21,22] and were sent as SMS text messages through mobile phones to a target audience of nonadherent individuals. *Asking* a question about a person’s intention to carry out a health behavior (CRC screening, in this case) in an SMS reminder was found to be more effective than an SMS reminder *stating* CRC screening was advised. Multimedia Appendix 1 displays the original experimental conditions.

This work is an account of recipient *responses* to the SMS reminders in less adherent population sectors invited for CRC screening. The study posed the following research questions (RQs):

RQ1: What characterized respondents to SMS reminders—demographic attributes, past screening participation, and the experimental condition?

RQ2: What does the response content reveal about attitudes and intentions regarding CRC screening?

RQ3: Are responses to reminders and their content associated with subsequent CRC screening participation?

RQ4: Does the response to the SMS mediate between the experimental condition and CRC screening?

## Methods

### Participants

**Figure 1:**
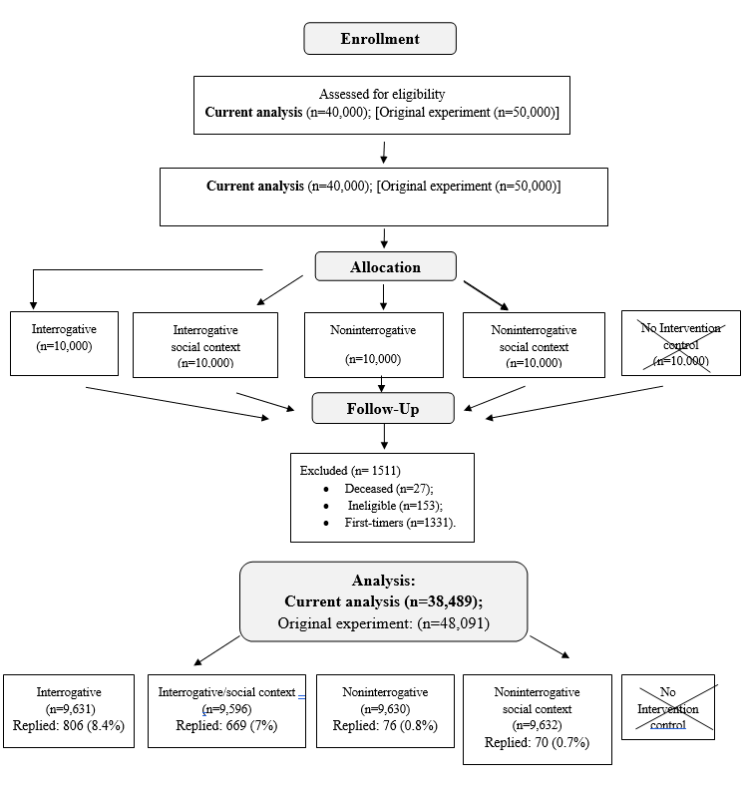
Flowchart of participants. The two boxes representing the control group are X-ed out, as the participants in the X-ed out boxes are not part of the current analyses.

In 2013, 50,000 Israeli women and men were routinely invited by mail to screen under the National Israeli Colorectal Cancer Early Detection program [4]. Participants were randomly assigned to 5 equal groups. Individuals in 4 groups received one of 4 SMS versions, while the fifth (control) group received none. This analysis focused on 40,000 addressees in the experimental groups (Figure 1). The Internal Review Board approval number for this study is as follows: 021–26513, 5.5.13 [4].

### Procedure

SMS text message reminders yielded responses that were analyzed as predictors of the subsequent fecal occult blood test (FOBT, recommended for individuals at average risk) performance. Demographic variables (age, gender, socioeconomic status, SES) and FOBT performance (past— 2004-2012; subsequent—within 6 months, 1 year, and 2 years following the SMS text message) were retrieved from the program’s computerized database.

### Materials

#### Short Message Service Text Messages Wording

The brief SMS text messages (122-135 characters) varied in grammatical form (interrogative/noninterrogative): “...do you intend to mail-order an FOBT kit and be tested?” or “...it is important to mail-order a kit and be tested,”), and social comparison of performing FOBT (“as others your age do”) [4]. Each version combined grammatical form with/without social comparison (Multimedia Appendix 1).

#### Responses to the Short Message Service Text Messages

In this study, responses to the SMS were open SMS text messages.

#### Demographic Characteristics

We retrieved demographic characteristics of participants from the Health Maintenance Organization database and included age, gender, and SES. The SES was determined by the address of the neighborhood clinic insured members attend; members of this Health Maintenance Organization generally attend primary health clinics located in their residential neighborhood. The SES of the clinic’s address was based on the classification by the Israeli Central Bureau of Statistics, which assigns an SES ranking to street addresses.

### Design

This was a prospective cohort experiment. Initially, the experimental conditions (grammatical form and social comparison) and background variables were independent variables, while participants’ responses constituted the dependent variable. In the content analysis stage, the responses, coded and grouped, served as the independent variable, while FOBT performance (past and subsequent) was the dependent variable.

### Data Analysis

First, respondents were characterized by experimental condition (grammatical form and social comparison), demographic attributes, and FOBT past performance (sporadic/never). A statistical test of main effects and interaction between grammatical form and social comparison was conducted on responding.

Responses to the SMS were coded for content and length. Two researchers (EN and LH) looked for underlying concepts in an open coding followed by axial coding [23], and labeled categories. The identified categories were grouped into 2 new variables as follows: attitude toward CRC screening and the intention to perform FOBT.

Next, using *χ*^2^ analyses, respondents were tested whether they held positive or negative attitudes toward CRC screening, and whether respondents’ intentions toward FOBT performance differed by demographic attributes, past FOBT screening rates, and experimental conditions. Of note, SMS responses were excluded only if they were illegible or if they were returned by ineligible respondents. In addition, the prospective association of the valence of the attitudes and intentions to undergo FOBT at 3 endpoints (at 6 months, 1 year, and 2 years) was examined, as well as the prospective association of SMS response to the reminders (Yes or No) with the undergoing of FOBT at the 3 endpoints mentioned above; all used a chi-square analysis.

Then, a multivariable analysis predicting FOBT screening after 6 months was conducted; the predictors were demographic variables, past (sporadic/never) FOBT performance, experimental conditions (interrogative/declarative), and response valence.

Finally, to test the mediational effect of the experimental condition (*X*) on the FOBT performance (*Y*) by responding to the SMS (*M*), we computed the appropriate indirect effect. To account for the binary nature of *M* and *Y*, we specified these variables as categorical and estimated a model using probit link with ordinal mediator; we followed the example presented in Table 8.26 of Muthén et al [24]. Furthermore, background variables (age, sex, SES, and past FOBT behavior) were included in the model to account for their possible confounding with *M* or *Y*.

This path model was estimated using the Mplus software [25]. See Multimedia Appendix 2 for model specifications, Mplus syntax code, the conceptual and statistical model, and detailed results of the path analysis model. As a robustness check, we also ran the analyses using the percentile bootstrapping method to account for potential nonnormality of our estimates [26].

## Results

### Responding to Short Message Service Text Messages (RQ1)

An SMS text message response was returned by 4.21% (1621/38,489) of the participants out of SMS recipients. As shown (Figure 1), 7.67% (1475/19,227) responses followed the interrogative conditions, while 0.76% (146/19,262) followed the declarative ones. The grammatical form had a significant effect on response (odds ratio [OR] 11.481, 95% CI 9.059 to 14.551; *P*<.001], while social comparison and the interaction between grammatical form and social comparison did not (OR 0.920, 95% CI 0.664 to 1.275; *P*<.617 and OR 0.892, 95% CI 0.633 to 1.258; *P*<.512, respectively). Social comparison conditions were collapsed in subsequent analyses.

A comparison between respondents and nonrespondents indicated that among respondents, there were significantly more women, individuals of a higher SES, and past FOBT sporadic performers. The response rate did not differ by age (Table 1).

### Content Analysis of Responses (RQ2)

The 1621 responses were read, repetitive themes were noted, and categories of responses were defined. Each response was coded accordingly. Researchers worked separately and mostly agreed; in a few cases with divergent judgments, a discussion led to an agreement. Table 2 presents categories and median length of the response field.

**Table 1:** A comparison between respondents and nonrespondents to the short message service text messages (N=38,489).

Characteristics		No response to SMS^a^ (N=36,868), n (%)	SMS respondents (N=1621), n (%)	*P* value
Gender, women		18,776 (50.92)	866 (53.42)	.049
Age, >60 years		18,462 (50.08)	791 (48.79)	.31
**Socioeconomic status**				<.001
	Low	9740 (26.42)	277 (17.09)	
	Medium	16,849 (45.70)	695 (42.87)	
	High	10,159 (27.56)	644 (39.73)	
**Past Fecal Occult Blood Test testing**				<.001
	Sporadic	9862 (26.75)	608 (37.51)	
	Never	27,006 (73.25)	1013 (62.49)	

^a^SMS: short message service.

**Table 2:** Short message service text message response categories (n=1621).

Content	Responses, n (%)	Code number	Median length of response field^a^ (IQR^b^)
Yes/OK	626 (38.6)	2	2 (2,6)
Please send me a kit	291 (18)	1	20 (14,31)
No/not interested	212 (13.1)	9	3 (2,7)
I underwent a colonoscopy	135 (8.3)	4	33 (26,43)
[illegible message]	88 (5.4)	7	10 (3,17)
I underwent the test under this program	85 (5.2)	3	25 (17,38)
I did not receive the invitation letter	63 (3.9)	10	18 (14,26)
I have a kit/will soon undergo the test	57 (3.5)	5	14 (9,24)
I have a question (regarding the test or CRC screening)	23 (1.4)	6	19 (11,43)
I was diagnosed with cancer (ie, ineligible for screening)	21 (1.3)	8	29 (17,44)
I underwent the test in a private clinic	13 (0.8)	12	39 (26,50)
Maybe (I’ll undergo the test) OR I might undergo the test	7 (0.4)	11	11 (7,27)

^a^In characters, including spaces.

^b^Interquartile range.

The median length of the SMS response field was informative—short for simple messages (#2 and #9), longer, higher variability in elaborate responses (#3, #4, and #12), explaining why respondents did not perform FOBT at this particular time.

The content categories that were identified were then grouped into 2 new variables, relevant to the QBE framework (focusing on participants’ intentions; see Multimedia Appendix 1).

The first grouped variable was “*Attitude* toward CRC screening.” Responses that implied support for CRC screening were coded as positive; these responses included: (1) explanations why respondents did not perform the test following this intervention (eg, already had undergone the test within the program, #3; or in a private clinic; #12; or underwent a colonoscopy, #4); (2) procedural questions (eg, asking for information on how to obtain an FOBT kit; #6); or (3) clear expressions of positive attitudes such as “yes,” “OK,” “please send me the kit,” “I will soon undergo the test” (such as in responses #1, #2, and #5), and leaning toward undergoing the test (“maybe”; #11). Thus, categories #1, #2, #3, #4, #5, #6, #11, and #12 were grouped as expressing a positive attitude. Categories #9 (“not interested”: “no”) and #10 (“did not receive an invitation”) were grouped as expressing a negative attitude. Note that already having taken the test (#3), having taken the test in a private clinic (#12), or having done another test (#4) express a positive position toward early detection of CRC (not necessarily toward FOBT).

The second grouped variable was “*Intention* to perform FOBT.” Categories #1, #2, #5, #6, and #11, with responses such as “please send me a kit” (#1), “yes,” “will soon do it” (#5), “I have a question” (#6), and “I may take the test” (#11) were interpreted as conveying an intention to screen. Conversely, categories #3, #4, #9, #10, and #12 where participants reported that they had undertaken screening (either colonoscopy or FOBT; #3 and #4, and #12) were uninterested (#9) or did not receive the invitation, were coded as expressing a negative intention. Most respondents (1237/1512, 81.8% participants) expressed a positive attitude toward CRC screening, and 62.6% (1004/1512) expressed an intention to screen using the FOBT modality.

A bivariate analysis showed that both positive attitudes and intentions toward CRC screening were associated with age (younger), and with past FOBT sporadic uptake (see Tables 3 and 4): individuals aged 50-60 years expressed more positive attitudes toward CRC screening than individuals aged >60 years (*χ*_*1*_^2^=7.4, *P*=.006), and an intention to undergo FOBT more than others aged >60 years (*χ*_*1*_^2^=25.2, *P*<.001). Similarly, past sporadic performers expressed a more positive attitude than the never tested (*χ*_*1*_^2^=17.8, *P*<.001) and showed more intent to undergo FOBT (*χ*_*1*_^2^=14.983, *P*<.001); the majority of “never-tested” participants expressed positive attitudes (735/936, 78.5%) and intentions (587/936, 62.7%). Attitudes regarding CRC screening and intentions to undergo FOBT were similar and nonsignificant by gender and SES. Finally, receivers of interrogative SMS were not different from receivers of declarative SMS in their attitudes, yet they expressed more intentions to undergo FOBT (944/1407, 67.1% vs 60/105, 57.1%, respectively; *χ*_*1*_^2^=4.3, *P*=.037).

**Table 3:** Attitudes toward colorectal cancer screening by participants’ background and past screening behavior (n=1512).

Characteristic		Positive (N=1237), n (%)	Negative (N=275), n (%)	*P* value
**Gender**				.58
	Women	670 (81.3)	154 (18.7)	
	Men	567 (82.4)	121 (17.6)	
**Age (years)**				.006
	50-60	661 (84.4)	122 (15.6)	
	Above 60	576 (79.0)	153 (21.0)	
**Socioeconomic status^a^**				.11
	Low	196 (77.8)	56 (22.2)	
	Medium	541 (83.7)	105 (16.3)	
	High	497 (81.6)	112 (18.4)	
**Past Fecal Occult Blood Test testing**				<.001
	Sporadic	502 (78.5)	74 (12.8)	
	Never	735 (78.5)	201 (21.5)	
**Experimental condition**				.582
	Declarative	88 (83.8)	17 (16.2)	
	Interrogative	1149 (81.7)	258 (18.3)	

^a^N=1507, owing to missing data.

**Table 4:** Intentions to undergo Fecal Occult Blood Test by participants’ background and past screening behavior (n=1512).

Characteristic		Yes (N=1004), n (%)	No (N=508), n (%)	*P* value
**Gender**				.67
	Women	551 (66.9)	273 (33.1)	
	Men	453 (65.8)	235 (34.2)	
**Age**				<.001
	50-60	566 (72.3)	217 (27.7)	
	Above 60	438 (60.1)	291 (39.9)	
**Socioeconomic status^a^**				.15
	Low	168 (66.7)	84 (33.3)	
	Medium	447 (69.2)	199 (30.8)	
	High	386 (63.4)	223 (36.6)	
**Past Fecal Occult Blood Test testing**				<.001
	Sporadic	417 (72.4)	159 (27.6)	
	Never	587 (62.7)	49 (37.3)	
**Experimental condition**				.04
	Declarative	60 (57.1)	45 (42.9)	
	Interrogative	944 (67.1)	463 (32.9)	

^a^N=1507, owing to missing data.

### Association Between Response Content and Undergoing Colorectal Cancer Screening (RQ3)

**Figure 2:**
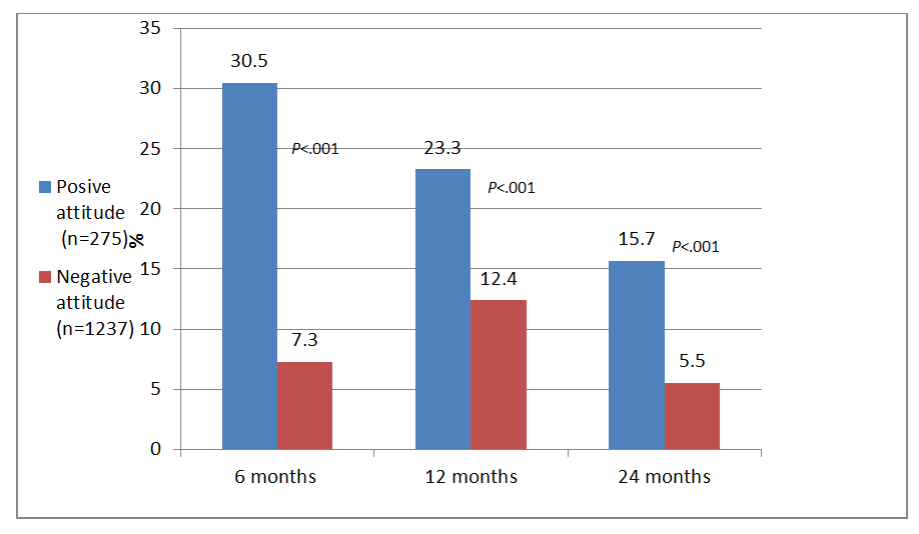
The Fecal Occult Blood Test uptake (at months) by attitude.

**Figure 3:**
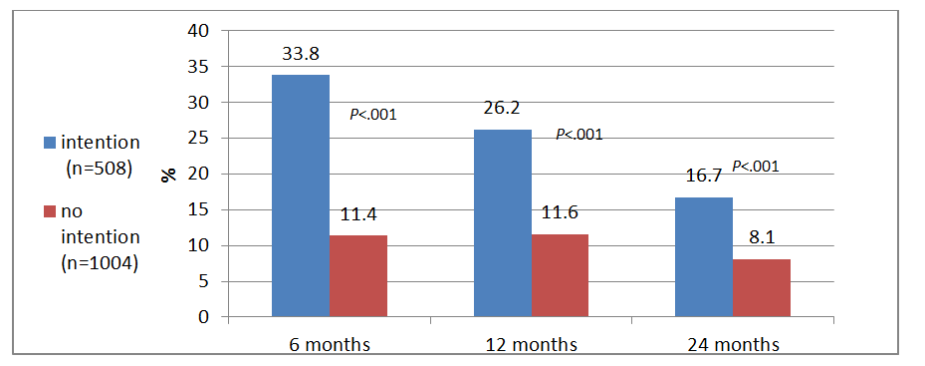
The Fecal Occult Blood Test uptake (at months) by intention.

Valence in the 2 grouped variables significantly distinguished between SMS respondents, as it was associated with undergoing FOBT in the *6 months* following sending of the SMS text messages—30.5% (377/1237) participants expressing a positive attitude toward CRC screening tested within the next 6 months, compared with 7.3% (20/275) who expressed a negative attitude (*χ*_*1*_^2^=62.5, *P*<.001). Participants who expressed no intention to undergo FOBT underwent the test significantly less than those who expressed an intention to test—11.4% (58/508), compared with 33.8% (339/1004), respectively (*χ*_*1*_^2^=87.0, *P*<.001). Similarly, 23.3% (288/1237) participants expressing a positive attitude toward CRC screening were tested *after 1 year*, compared with 12.4% (34/275) who expressed a negative attitude (*χ*_*1*_^2^=16.0, *P*<.001). Participants who expressed no intention to undergo FOBT underwent the test significantly less than those who expressed an intention to test—11.6% (59/508), compared with 26.2% (263/1004), respectively (*χ*_*1*_^2^=42.8, *P*<.001). *Two years* following the intervention, 15.7% (194/1237) participants who had expressed a positive attitude toward CRC screening were tested, compared with 5.5% (15/275) who had expressed a negative attitude (*χ*_*1*_^2^=19.8, *P*<.001). Participants who had expressed no intention to undergo FOBT underwent the test significantly less than those who expressed an intention to test—8.1% (41/508), compared with 16.7% (168/1004), respectively (*χ*_*1*_^2^=21.3, *P*<.001). Figures 2 and 3 display screening at 6, 12, and 24 months following reminders by attitude and intentions.

**Figure 4:**
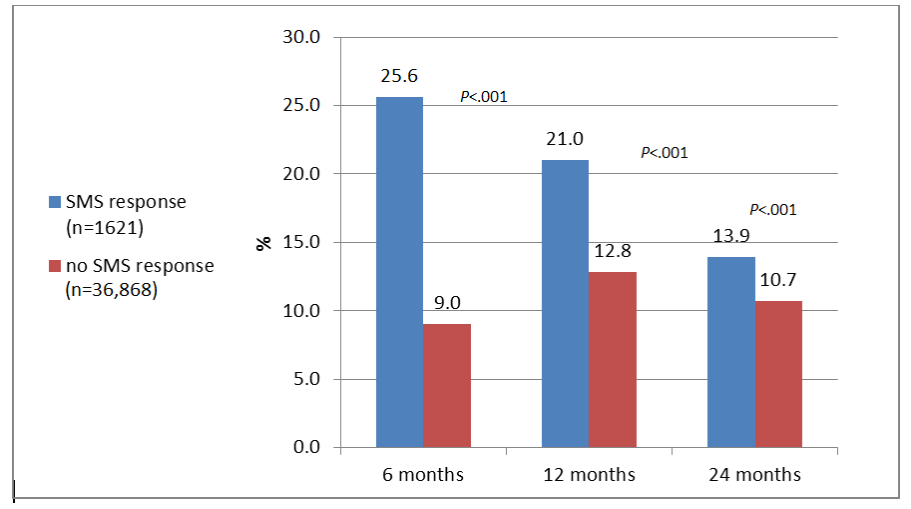
The Fecal Occult Blood Test uptake (at months) by response to short message service (SMS) text message.

Though the interrogative conditions yielded 10 times more responses than the declarative conditions, participants who chose to respond, across experimental conditions, underwent FOBT more than nonrespondents after 6 months (415/1621, 25.60% vs 3322/36,868, 9.01%; *χ*_*1*_^2^=487.5, *P*<.001; Figure 4). The difference was significant after 1 year (340/1621, 20.97% vs 4711/36,868, 12.78%; *χ*_*1*_^2^=91.50, *P*<.001), and even after 2 years (225/1621, 13.88% vs 3924/36,868, 10.64%; *χ*_*1*_^2^=16.92, *P*<.001).

Then, a multivariable logistic regression on respondents (n=1507) was carried out, with FOBT performance after 6 months as the dependent variable. The predictors were demographic variables, past (sporadic/never) FOBT performance, the 2 grouped variables, *attitude* and *intention*, and the experimental condition. Age (older), past sporadic FOBT performance, attitude, and intention to test expressed in the SMS text message response had a significant effect (OR 1.421, 95% CI 1.097 to 1.840; *P*=.008; OR 3.271, 95% CI 2.540 to 4.213; *P*<.001; OR 2.166, 95% CI 1.204 to 3.894; *P*=.010; OR 2.817, 95% CI 1.909 to 4.156; *P*<.001, respectively).

### Mediation Analysis: The Path Between the Experimental Condition (Short Message Service Text Message Type), Responding and Screening (RQ4)

The indirect effect (ie, the total natural indirect effect) of *X*, the experimental manipulation, on *Y* through *M*, was positive and significant (estimate=0.005, *P*<.000), while the pure natural direct effect was insignificant (estimate=–0.004, *P*=.083). The percentile bootstrapping method (with 1000 replicates) yielded similar results—the total natural indirect effect was 0.005 (95% CI 0.004 to 0.006), while the pure natural direct effect was –0.004 (95% CI –0.008 to 0.001). Figure 2 and Table 1 in Multimedia Appendix 2 present detailed results.

## Discussion

### Principal Findings and Comparison With Prior Work

This analysis addressed responses to mobile phone SMS reminders to enhance the CRC screening participation among a nonadherent sector of the target population. Spontaneous, open SMS text message responses returned to the screening team uncovered another aspect of participants’ characteristics, as related to their intention and their subsequent screening uptake.

SMS text message respondents in a nonadherent sector of the target population provided the following 3 indicators: the act of sending back an SMS text message, its content, and engaging in CRC screening. The main findings, discussed in this order, were as follows: (1) interrogative SMS text messages yielded more responses than did typical, declarative reminders (RQ1); (2) the act of responding was predictive of subsequent screening (after 6 months, 1 and 2 years; RQ3); (3) the response content (valence; RQ2) was predictive, across conditions, of subsequent screening (at same time-points; RQ3); (4) in a multivariate analysis, the response valence was predictive of subsequent screening, while experimental conditions were not (ie, respondents across conditions displayed similar screening rates; RQ3); (5) response to the SMS reminders positively and significantly mediated between the experimental condition and CRC screening; and (6) age was related to the response content and subsequent FOBT screening; previous FOBT performance was related to repeating this behavior (RQ1).

The interrogative conditions in this study yielded 10-fold more responses than the declarative conditions, and the positive responses were associated with the target behavior. Furthermore, responding to the message mediated the effect of the experimental condition on the FOBT performance. This joins previous findings in the health domain [27,28] and further attests to the motivating power of asking questions about the intention to enact health behaviors. Moreover, the linguistic form (interrogative vs declarative) and the responses it generated afforded a rare glimpse into the “introspective self-talk” [29], which theorists posited as enhancing intrinsic motivation. As opposed to questions, statements rarely elicited an internal dialogue, as manifested in more intentions expressed by interrogative SMS receivers than by declarative SMS receivers.

The content of the responses covered the entire range from “yes” and “send me the kit” to explanations why one would not perform the targeted behavior, including “not interested” or “no.” The longer responses (ie, extended field length) comprised explanations why respondents would not perform the behavior; this was participants’ way to share ideas from their introspective self-talk. The circumstances described ranged from a cancer diagnosis (ineligible to test for the early detection of this disease) to having already screened under a different modality, timing, or health provider.

What is more important for FOBT screening in an SMS reminder intervention—the act of responding or the content of the response? This study could not address this question directly, as there were no content data for nonrespondents. The multivariate analysis provided indirect indications that, among respondents, content (attitudes and intentions) was predictive of screening, while the experimental condition was not. While the content of the “introspective dialogue” matters, it is activated by questions, suggesting a possible mechanism behind the advantage of interrogative reminders.

The respondents in the younger category (≤60 years) were more positive toward CRC screening, expressing an intention to screen more frequently than respondents in the older age category (60+). The intention to conduct a recommended health behavior was a strong predictor of carrying out the behavior [30]. Nevertheless, the older age group screened significantly more than the younger age group within the next 6 months, possibly affected by their previous higher screening rates. Indeed, a gap is apparent between the attitude and intention, on the one hand, and the behavior, on the other; more work is needed to promote screening among younger individuals.

To date, studies have documented adherent individuals to CRC screening with FOBT—as consistently being women and older individuals worldwide [31-33] as well as in Israel [4,34,35]. Increasing FOBT uptake among men and younger age groups (50-60 years) of the target population has been the central aim of screening program organizers for some time. The current findings regarding the efficacy of the interrogative SMS text messages reaching the younger age group, both women and men as well as their expression of positive attitudes and intentions, are evidence that a nonadherent sector of the target population for CRC screening has been reached by the interrogative SMS text messages. The use of SMS “filtered” respondents, inspiring feedback from those who, thus far, have not (regularly, or at all) been involved in CRC screening. This has not yet materialized to a screening behavior among individuals in the younger age group, epitomizing the intention–behavior gap [36].

Undergoing FOBT once is a predictor of repeating this annually recommended health behavior [37]. Including cycle screenees who have never tested and those who have undergone the test irregularly in the screening may contribute to their future routine screening. The more individuals repeat screening, the more this health behavior becomes part of their lifestyle [38]. The 2-year CRC screening follow-up of an SMS reminder, not reported previously, may be a chain-reaction triggered by the SMS, in which participants entered the screening cycle following the reminder, remaining “in the loop” for years to come.

To date, few studies have addressed the unique characteristic of SMS immediacy combined with social contact [39], particularly the space for dialogue carved out by the interrogative wording. Such a dialogue is central to health care and supporting patients in taking recommended action to enhance their health. The technique is scalable to population-level health interventions. Response content and respondents’ characteristics and screening patterns highlight a complex, dynamic aspect of “nonadherence” to CRC screening, which program administrators could address; for example, by preprogramming responses sent as a reply to frequently used comments and sharing patient concerns/questions (a mere 1.4% of the responses) with the attending physician.

### Strengths and Limitations

The strengths of this field experiment are as follows [4]: an objective outcome measure, a large sample size, and the mHealth method: simple, inexpensive, and parsimonious. The additional 2-year follow-up of a single, short, interrogative SMS reminder, to engaging in health behavior (CRC screening) years later, attests to the impact of the technique’s bidirectionality. The comparison of the response content, in receivers of question- vs statement-mode reminders, also pointed, in addition to the higher yield of responses to questions, to a possible explanation. Potentially wide, scalable [40] applications to enhance health behaviors are implied here, which could be used in everyday practice, replacing declarative recommendations—asking patients to predict what they would do, “Do you intend to...?” [41] activates the introspective self-talk [29], which is more effective than “you need to do this.” The interrogative wording has rarely been used in SMS text messages. Even though SMS text messages are used abundantly, an examination of alternative wordings has not yet been published. Finally, the mediating effect of the SMS response was indicated using a state-of-the-art statistical technique of mediation analysis.

Study limitations include the lack of evidence that participants read the message. Second, the organizational signature concluding the message may have been less effective than if the attending physician had signed it. In addition, this study did not directly address mechanisms underlying QBE, which may be the goal of further work. Furthermore, implementing the recommendation to *ask* rather than *tell* in interpersonal encounters in the health care setting may seem challenging for established professionals. Finally, the analysis is limited by the lack of data on potentially important confounders such as digital literacy and health status.

Future studies may examine the routine use of SMS interrogative reminders to encourage FOBT kit holders who procrastinate in undergoing testing or supporting other behavioral modifications such as appointment attendance or medication adherence. The strength of posing questions stems from the internal dialogue which follows; interviews with respondents to SMS reminders may shed light on this phenomenon and possibly shape the design of future studies that will attempt to tease the effect of responding apart from the effect of the response content.

### Conclusions

SMS interrogative reminders to undergo CRC screening with FOBT have had a long-term effect on sectors in the target population who rarely tested previously, reaching men and younger adults, who expressed positive attitudes toward screening and intentions to test. Medical recommendations, phrased interrogatively, may be more effective than statements. This work provides evidence for this also in the mHealth arena; asking patients may promote behavior change in face-to-face encounters in the clinic and other patient communications.

## Appendix

Multimedia Appendix 1 Text messages.

Multimedia Appendix 2 Mediation analysis.

## Abbreviations

CRC: colorectal cancer
FOBT: Fecal Occult Blood Test
mHealth: mobile health
OR: odds ratio
QBE: question-behavior-effect
RQ: research question
SES: socioeconomic status
SMS: short message service

## References

[1] Hiatt R, Wardle J, Vernon S, Austoker J, Bistanti L, Fox S, Gnauck R, Iverson D, Mandelson M, Reading D, Smith R (2005). Workgroup IV: public education. UICC International Workshop on Facilitating Screening for Colorectal Cancer, Oslo, Norway (29 and 30 June 2002). Ann Oncol.

[2] Baker DW, Brown T, Buchanan DR, Weil J, Balsley K, Ranalli L, Lee JY, Cameron KA, Ferreira MR, Stephens Q, Goldman SN, Rademaker A, Wolf MS (2014). Comparative effectiveness of a multifaceted intervention to improve adherence to annual colorectal cancer screening in community health centers: a randomized clinical trial. JAMA Intern Med.

[3] Calderwood Audrey H, Roy Hemant K (2013). Increasing colorectal cancer screening adherence: comment on “A randomized comparison of print and web communication on colorectal cancer screening”. JAMA Intern Med.

[4] Hagoel L, Neter E, Stein N, Rennert G (2016). Harnessing the question-behavior effect to enhance colorectal cancer screening in an mHealth experiment. AJPH.

[5] Hillebrand F (2010). Who invented SMS?. Short Message Service (SMS): The Creation of Personal Global Text Messaging.

[6] Hillebrand Ff (2010). Conclusions. Short Message Service (SMS): The Creation of Personal Global Text Messaging.

[7] (2013). Nielsen.

[8] Epley R http://www.tech-stress.com/1-5-how-the-smartphone-is-viewed-by-different-generations/.

[9] Starr K, McPherson G, Forrest M, Cotton SC (2015). SMS text pre-notification and delivery of reminder e-mails to increase response rates to postal questionnaires in the SUSPEND trial: a factorial design, randomised controlled trial. Trials.

[10] Boksmati N, Butler-Henderson K, Anderson K, Sahama T (2016). The Effectiveness of SMS Reminders on Appointment Attendance: a Meta-Analysis. J Med Syst.

[11] Guy R, Hocking J, Wand H, Stott S, Ali H, Kaldor J (2012). How effective are short message service reminders at increasing clinic attendance? A meta-analysis and systematic review. Health Serv Res.

[12] Kampman C, Koedijk F, Driessen-Hulshof H, Hautvast J, van den Broek I (2016). Retesting young STI clinic visitors with urogenital Chlamydia trachomatis infection in the Netherlands; response to a text message reminder and reinfection rates: a prospective study with historical controls. Sex Transm Infect.

[13] Park LG, Howie-Esquivel J, Dracup K (2014). A quantitative systematic review of the efficacy of mobile phone interventions to improve medication adherence. J Adv Nurs.

[14] Park LG, Beatty A, Stafford Z, Whooley MA (2016). Mobile Phone Interventions for the Secondary Prevention of Cardiovascular Disease. Prog Cardiovasc Dis.

[15] Chow C, Redfern J, Hillis G, Thakkar J, Santo K, Hackett M, Jan Stephen, Graves Nicholas, de Keizer Laura, Barry Tony, Bompoint Severine, Stepien Sandrine, Whittaker Robyn, Rodgers Anthony, Thiagalingam Aravinda (2015). Effect of Lifestyle-Focused Text Messaging on Risk Factor Modification in Patients With Coronary Heart Disease: A Randomized Clinical Trial. JAMA.

[16] Car NJ, Christen EW, Hornbuckle JW, Moore GA (2012). Using a mobile phone Short Messaging Service (SMS) for irrigation scheduling in Australia – Farmers’ participation and utility evaluation. Computers and Electronics in Agriculture.

[17] Revere D, Calhoun R, Baseman J, Oberle M (2015). Exploring bi-directional and SMS messaging for communications between Public Health Agencies and their stakeholders: a qualitative study. BMC Public Health.

[18] Walsh E, Brinker J (2016). Short and Sweet? Length and Informative Content of Open-Ended Responses using SMS as a Research Mode. J Comput Commun?.

[19] Sequist TD, Zaslavsky AM, Colditz GA, Ayanian JZ (2011). Electronic patient messages to promote colorectal cancer screening: a randomized controlled trial. Arch Intern Med.

[20] Rodrigues AM, O'Brien Nicola, French DP, Glidewell L, Sniehotta FF (2015). The question-behavior effect: genuine effect or spurious phenomenon? A systematic review of randomized controlled trials with meta-analyses. Health Psychol.

[21] Mahler HIM, Kulik JA, Gerrard M, Gibbons FX (2010). Effects of upward and downward social comparison information on the efficacy of an appearance-based sun protection intervention: a randomized, controlled experiment. J Behav Med.

[22] Lipkus I, Klein W (2006). Effects of communicating social comparison information on risk perceptions for colorectal cancer. J Health Commun.

[23] Strauss AL (1987). Qualitative Analysis Soc Scientists.

[24] Muthén B, Muthén L, Asparouhov T (2017). Regression and mediation analysis using Mplus.

[25] Muthén L, Muthén BO (2017). Mplus Version 8 User's Guide.

[26] Mackinnon DP, Lockwood CM, Williams J (2004). Confidence Limits for the Indirect Effect: Distribution of the Product and Resampling Methods. Multivariate Behav Res.

[27] Godin G, Sheeran P, Conner M, Delage G, Germain M, Bélanger-Gravel Ariane, Naccache H (2010). Which survey questions change behavior? Randomized controlled trial of mere measurement interventions. Health Psychol.

[28] Godin G, Bélanger-Gravel A, Vézina-Im L, Amireault S, Bilodeau A (2012). Question-behaviour effect: a randomised controlled trial of asking intention in the interrogative or declarative form. Psychol Health.

[29] Senay I, Albarracín D, Noguchi K (2010). Motivating goal-directed behavior through introspective self-talk: the role of the interrogative form of simple future tense. Psychol Sci.

[30] Sheeran P, Maki A, Montanaro E, Avishai-Yitshak A, Bryan A, Klein WMP, Miles E, Rothman AJ (2016). The impact of changing attitudes, norms, and self-efficacy on health-related intentions and behavior: A meta-analysis. Health Psychol.

[31] Meissner HI, Breen N, Klabunde CN, Vernon SW (2006). Patterns of colorectal cancer screening uptake among men and women in the United States. Neuropharmacology.

[32] Javanparast S, Ward P, Young G, Wilson C, Carter S, Misan G, Cole S, Jiwa M, Tsourtos G, Martini A, Gill T, Baratiny G, Matt MA (2010). How equitable are colorectal cancer screening programs which include FOBTs? A review of qualitative and quantitative studies. Prev Med.

[33] Klabunde C, Blom J, Bulliard J, Garcia M, Hagoel L, Mai V, Patnick J, Rozjabek H, Senore C, Törnberg S (2015). Participation rates for organized colorectal cancer screening programmes: An international comparison. J Med Screen.

[34] Ore L, Hagoel L, Lavi I, Rennert G (2001). Screening with faecal occult blood test (FOBT) for colorectal cancer: assessment of two methods that attempt to improve compliance. Eur J Cancer Prev.

[35] Neter E, Stein N, Barnett-Griness O, Rennert G, Hagoel L (2014). From the bench to public health: population-level implementation intentions in colorectal cancer screening. Am J Prev Med.

[36] McEachan RR, Conner M, Taylor NJ, Lawton RJ (2011). Prospective prediction of health-related behaviours with the theory of planned behaviour: A meta-analysis. Health Psychology Review.

[37] Conner M, Norman P, Bell R (2002). The theory of planned behavior and healthy eating. Health Psychol.

[38] Hagoel L, Ore L, Neter E, Shifroni G, Rennert G (1999). The gradient in mammography screening behavior: a lifestyle marker. Soc Sci Med.

[39] Riley WT, Rivera DE, Atienza AA, Nilsen W, Allison SM, Mermelstein R (2011). Health behavior models in the age of mobile interventions: are our theories up to the task?. Transl Behav Med.

[40] Thakkar J, Kurup R, Laba T, Santo K, Thiagalingam A, Rodgers A (2016). Mobile Telephone Text Messaging for Medication Adherence in Chronic Disease. JAMA Intern Med.

[41] Wilding S, Conner M, Sandberg T, Prestwich A, Lawton R, Wood C (2016). The question-behaviour effect: A theoretical and methodological review and meta-analysis. Eur Rev Soc Psychol.

