# Supplementary material for: Better Ask Than Tell: Responses to mHealth Interrogative Reminders and Associations With Colorectal Cancer Screening Subsequent Uptake in a Prospective Cohort Intervention
Source: JMIR Mhealth Uhealth. 2019 Jan 21;7(1):e9351. doi: 10.2196/mhealth.9351 (PMC6360382; doi:10.2196/mhealth.9351)
Supplement: Multimedia Appendix 1 [file mhealth_v7i1e9351_app1.pdf]

Appendix 1

Text messages used in the parent study

Experimental condition		Phrasing of text sent
1.	Interrogative	"Following the invitation to screen for CRC, recently sent to you, do you intend to mail-order an FOBT kit and be tested? At your service, CHS"
2.	Interrogative + social context	"Following the invitation to screen for CRC, recently sent to you, do you intend to mail-order an FOBT kit and be tested, as others your age do? At your service, CHS"
3.	Non-interrogative	"Following the invitation to screen for CRC, recently sent to you -- it is important to mail-order a kit and be tested. At your service, CHS"
4.	Non-interrogative + social context	"Following the invitation to screen for CRC, recently sent to you -- people your age mail-order an FOBT kit and undergo the test. At your service, CHS"
5.	No intervention	None
