# Supplementary material for: Better Ask Than Tell: Responses to mHealth Interrogative Reminders and Associations With Colorectal Cancer Screening Subsequent Uptake in a Prospective Cohort Intervention
Source: JMIR Mhealth Uhealth. 2019 Jan 21;7(1):e9351. doi: 10.2196/mhealth.9351 (PMC6360382; doi:10.2196/mhealth.9351)
Supplement: Multimedia Appendix 2 [file mhealth_v7i1e9351_app2.pdf]

Appendix 2

Mediation analysis: Syntax, conceptual model, statistical model and results of the path model

Mplus syntax:

```
<

variable:

    names = id_num X_SMS_TY C4_PreFO M_SMS_re Y_FO_per

            C1_Female C2_age60N C3_SES_2 C3_SES_3;

    categorical = M_SMS_re Y_FO_per;! binary outcome and mediator

    usevar =  X_SMS_TY C4_PreFO M_SMS_re Y_FO_per

            C1_Female C2_age60N C3_SES_2 C3_SES_3;,


MISSING ARE ALL(-1234);


Analysis:

        estimator = ml;

        link = probit;


model:

    M_SMS_re on X_SMS_TY

      C1_Female C2_age60N C3_SES_2 C3_SES_3 C4_PreFO;


    Y_FO_per on X_SMS_TY M_SMS_re

      C1_Female C2_age60N C3_SES_2 C3_SES_3 C4_PreFO;



model indirect:

  Y_FO_per  ind M_SMS_re X_SMS_TY;


output:

        tech1 tech8 sampstat patterns cinterval;
```

Figure 1
The conceptual model

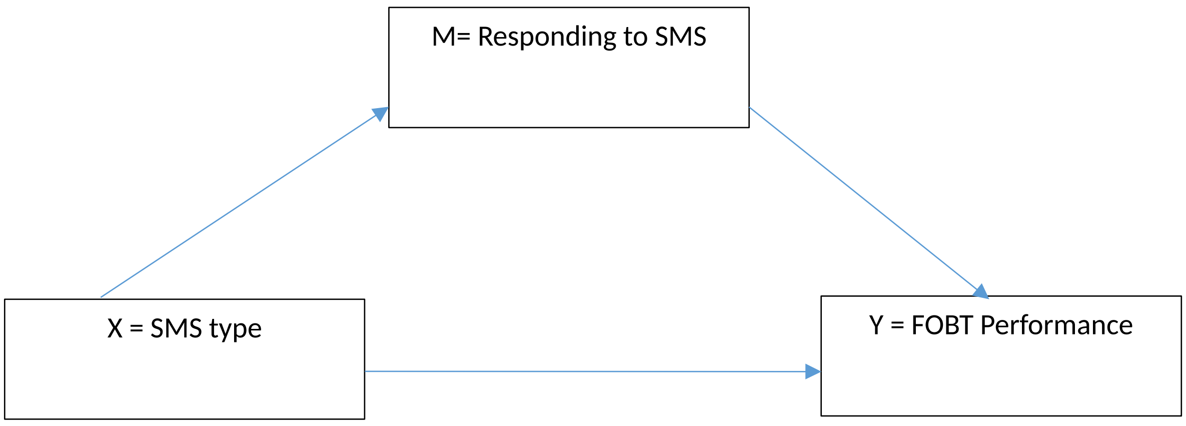

Figure 2
The statistical model

Main Results of the Path Model (Unstandardized Coefficients with Standard Errors in

(Parentheses; Control Variables are not Depicted; *** p < .001

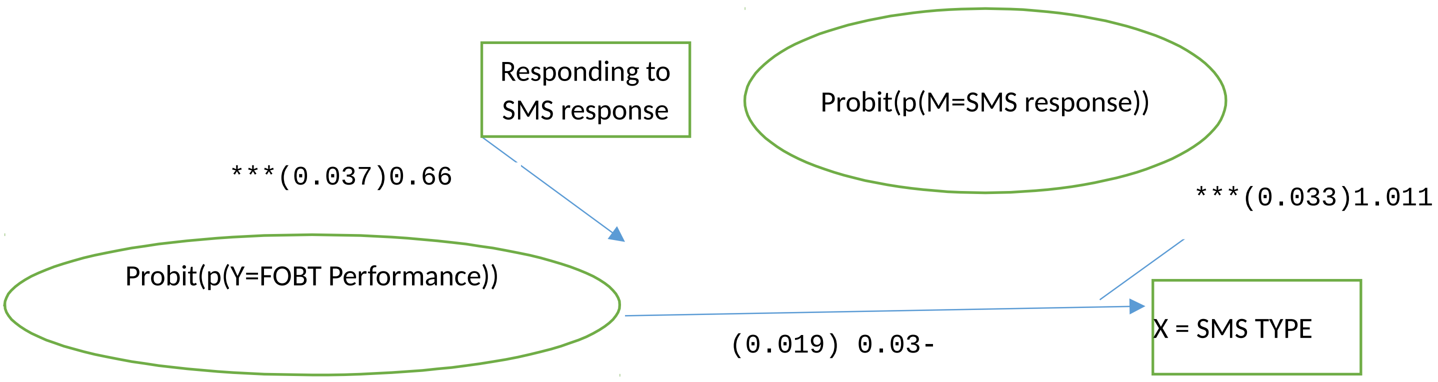

Table 1
Results of the Path Analysis Model

	Responding to SMS		FOBT Performance	
	B	SE	B	SE
Gender, female [a]	0.029	0.025	0.073***	0.018
Age, b	-0.080***	0.025	-0.029	0.019
SES, c	0.183***	0.033*	-0.024	0.022
SES, d	0.398***	0.035	-0.102***	0.025
Past FOBT testing e	0.263***	0.027	0.663***	0.019
SMS Type	1.011***	0.033	-0.032	0.019
Responding to SMS	--		0.663***	0.037

Note. N = 38364; unstandardized coefficients are shown; a 0 = male, 1 = female; b 0 = age under or equal to 60, 1 = age above 60; c 0 = Low or high SES, 1 = medium SES; d 0 = Low or medium SES, 1 = high SES; e 0 = No Previous FOBT, 1 = Yes Previous FOBT;
.*p* < .05; ** *p* < .01 *
